# Cost-benefit of hospitalization compared with outpatient care for pregnant women with pregestational and gestational diabetes or with mild hyperglycemia, in Brazil

**DOI:** 10.1590/S1516-31802012000100004

**Published:** 2012-02-14

**Authors:** Ana Claudia Molina Cavassini, Silvana Andréa Molina Lima, Iracema Mattos Paranhos Calderon, Marilza Vieira Cunha Rudge

**Affiliations:** I Nurse, Municipal Authority of Botucatu, Botucatu, São Paulo, Brazil.; II MD, PhD. Assistant Professor, Department of Nursing, Hospital das Clínicas (HC), Faculdade de Medicina de Botucatu (FMB), Universidade Estadual Paulista (Unesp), Botucatu, São Paulo, Brazil.; III MD, PhD. Adjunct Professor, Department of Gynecology and Obstetrics, Hospital das Clínicas (HC), Faculdade de Medicina de Botucatu (FMB), Universidade Estadual Paulista (Unesp), Botucatu, São Paulo, Brazil.; IV MD, PhD. Titular Professor, Department of Gynecology and Obstetrics and Pro-Rector of Postgraduate Programs, Universidade Estadual Paulista (Unesp), Botucatu, São Paulo, Brazil.

**Keywords:** Hospital costs, Gestational diabetes, Cost-benefit analysis, Health care costs, Costs and cost analysis, Custos hospitalares, Diabetes gestacional, Análise custo-benefício, Custos de cuidados de saúde, Custos e análise de custo

## Abstract

**CONTEXT AND OBJECTIVE:**

Pregnancies complicated by diabetes are associated with increased numbers of maternal and neonatal complications. Hospital costs increase according to the type of care provided. This study aimed to estimate the cost-benefit relationship and social profitability ratio of hospitalization, compared with outpatient care, for pregnant women with diabetes or mild hyperglycemia.

**STUDY DESIGN:**

This was a prospective observational quantitative study conducted at a university hospital. It included all pregnant women with pregestational or gestational diabetes, or mild hyperglycemia, who did not develop clinical intercurrences during pregnancy and who delivered at the Botucatu Medical School Hospital (Hospital das Clínicas, Faculdade de Medicina de Botucatu, HC-FMB) of Universidade Estadual de São Paulo (Unesp).

**METHODS:**

Thirty pregnant women treated with diet were followed as outpatients, and twenty treated with diet plus insulin were managed through frequent short hospitalizations. Direct costs (personnel, materials and tests) and indirect costs (general expenses) were ascertained from data in the patients’ records and the hospital’s absorption costing system. The cost-benefit was then calculated.

**RESULTS:**

Successful treatment of pregnant women with diabetes avoided expenditure of US$ 1,517.97 and US$ 1,127.43 for patients treated with inpatient and outpatient care, respectively. The cost-benefit of inpatient care was US$ 143,719.16, and outpatient care, US$ 253,267.22, with social profitability of 1.87 and 5.35, respectively.

**CONCLUSION:**

Decision-tree analysis confirmed that successful treatment avoided costs at the hospital. Cost-benefit analysis showed that outpatient management was economically more advantageous than hospitalization. The social profitability of both treatments was greater than one, thus demonstrating that both types of care for diabetic pregnant women had positive benefits.

## RESUMO

**CONTEXTO E OBJETIVO:**

Gestações complicadas pelo diabetes estão associadas com aumento de complicações maternas e neonatais. Os custos hospitalares aumentam de acordo com a assistência prestada. O objetivo foi calcular o custo-benefício e a taxa de rentabilidade social da hospitalização comparada ao atendimento ambulatorial em gestantes com diabetes ou com hiperglicemia leve.

**DESENHO DO ESTUDO:**

Estudo prospectivo, observacional, quantitativo, realizado em hospital universitário, sendo incluídas todas as gestantes com diabetes pregestacional e gestacional ou com hiperglicemia leve que não desenvolveram intercorrências clínicas na gestação e que tiveram parto no Hospital das Clínicas, Faculdade de Medicina de Botucatu, Universidade Estadual Paulista (HC-FMB-Unesp).

**MÉTODOS:**

Trinta gestantes tratadas com dieta foram acompanhadas em ambulatório e 20 tratadas com dieta e insulina foram abordadas com hospitalizações curtas e frequentes. Foram obtidos custos diretos (pessoal, material e exames) e indiretos (despesas gerais) a partir de dados contidos no prontuário e no sistema de custo por absorção do hospital e posteriormente calculado o custo-benefício.

**RESULTADOS:**

O sucesso do tratamento das gestantes diabéticas evitou o gasto de US$ 1.517,97 e US$ 1.127,43 para pacientes hospitalizadas e ambulatoriais, respectivamente. O custo-benefício da atenção hospitalizada foi US$ 143.719,16 e ambulatorial, US$ 253.267,22, com rentabilidade social 1,87 e 5,35 respectivamente.

**CONCLUSÃO:**

A análise “árvore de decisão” confirma que o sucesso dos tratamentos elimina custos no hospital. A relação custo-benefício indicou que o tratamento ambulatorial é economicamente mais vantajoso do que a hospitalização. A rentabilidade social de ambos os tratamentos foi maior que 1, indicando que ambos os tipos de atendimento à gestante diabética têm benefício positivo.

## INTRODUCTION

During their reproductive years, women have higher medical expenditure than men.^1^ Such expenditure is mostly related to pregnancy care, childbirth and the ensuing complications. Pregnancy and childbirth costs include the cost of prenatal care, hospitalizations and neonatal care. These costs increase with the need for hospitalization during pregnancy and length of hospital stays, and according to the mode of delivery. Adverse gestational outcomes also contribute towards substantially higher costs.^2^^,^^3^

Diabetes and mild hyperglycemia during pregnancy increase the costs of prenatal care. Prenatal hospitalization, an approach used in more severe difficult-to-control cases, makes costs even higher. Over the past two decades, improvements in maternal and perinatal outcomes from diabetic pregnancies have been well documented.^4^ Moreover, it has also been demonstrated that pregnant women with mild hyperglycemia, i.e. with a normal 100-g glucose tolerance test (GTT) but an abnormal glycemic profile, have perinatal outcomes comparable to those of diabetic women and should, therefore, receive comparable treatment.^5^^,^^6^ The principal factor responsible for this is the need for strict glycemic control to maintain blood glucose levels within the normal range throughout pregnancy.^7^ To obtain such control, these women are treated with diet and/or insulin therapy, and followed up on an outpatient basis or subjected to frequent short hospitalizations. The financial and social advantages of outpatient management are intuitively well understood, but a comprehensive comparison of maternal and perinatal outcomes with those of hospitalization is necessary.^8^

There are a variety of approaches to cost analysis: absorption costing, procedure cost, activity-based cost, etc. These methods make it possible to produce full or partial assessments.^9^ Cost minimization, cost-effectiveness, and cost-benefit analyses are the main types of full analysis. In turn, partial analyses measure the costs attributable to a particular disease and compare costs. Cost-benefit analysis measures costs and outcomes in monetary units. Because assigning monetary values to pertinent outcomes and human life is difficult, this type of analysis has not often been reported in the literature.

In order to determine the cost-benefit relationship of a treatment protocol, it is necessary to assess its impact on health improvement and the amount of money saved using a specific protocol, or to detail the health benefits achieved at a reasonable cost.^10^^,^^11^^,^^12^

Thus, there are some issues that should be taken into consideration. Firstly, treatment expenditure needs to be assessed. Secondly, it has to be borne in mind that some healthcare interventions initially increase the costs with the intent of subsequently providing a monetary saving. Thirdly, some costs are usually underestimated and there are difficulties in assigning values to them, such as the costs relating to pain and the distress of family members, among others.^13^

In an era of increasing healthcare costs, understanding the economics of medical care has become yet another requirement for the practice of medicine.^14^ However, there are only a few reports on the costs of diabetic pregnancies. More recent studies have assessed the cost-effectiveness of approaches towards diagnosing and treating gestational diabetes.^8^^,^^15^^,^^16^^,^^17^ In turn, other earlier studies evaluated the costs and benefits of treating diabetic pregnant women,^18^ and different strategies for assessing the quality of glycemic control^19^ or monitoring blood glucose.^20^ In a review of the literature, Brandle and Herman^2^ concluded that the cost-effectiveness of different types of treatment for gestational diabetes still need to be unequivocally demonstrated.

Analysis on inpatient insulin treatment for pregnant women with diabetes has shown that this therapeutic strategy is effective. Despite increasing the cost of gestational diabetes treatment, this can save the additional costs of possible complications.^17^ A cost-consequence analysis on hospital treatment with insulin among 100 women with gestational diabetes revealed that perinatal morbidity and mortality were reduced.^16^

Treatment for diabetes in pregnant women, including different conventional or intensive therapeutic strategies such as outpatient management or hospitalization, increases healthcare expenditure.^2^ These high maternal and infant costs incurred during the short period of pregnancy must be analyzed from the perspective of health economics. Therefore, the effectiveness of interventions should be analyzed and demonstrated.

The fundamental principles behind cost-benefit analysis reflect the need to determine an efficient allocation of resources. Cost-benefit analysis plays a leading role in investigating the usefulness of large-scale projects or interventions. Projects, investments or interventions with positive benefits are candidates for application of such analysis.^13^

In Brazil, the cost-benefit relationship of care for pregnant women with diabetes or mild hyperglycemia remains unreported. In these cases, two treatment strategies are used: frequent short hospitalizations or outpatient management. Comparing the costs of these strategies with the maternal and perinatal benefits is necessary.

## OBJECTIVES

The purpose of this study was to determine the cost-benefit relationship and social profitability ratio of hospitalization compared with outpatient management, for pregnant women with pregestational and gestational diabetes or mild hyperglycemia who attended the Center for Investigation of Perinatal Diabetes (Centro de Investigação do Diabete Perinatal, CIDP) of Botucatu Medical School Hospital (Hospital das Clínicas, Faculdade de Medicina de Botucatu, HC-FMB), Universidade Estadual Paulista (Unesp).

### The specific goals were as follows:

- To determine the maternal and perinatal criteria that define successful treatment of pregestational and gestational diabetes and mild hyperglycemia;
- To build a decision tree for each type of treatment for pregestational and gestational diabetes and mild hyperglycemia;
- To estimate the cost-benefit relationships of hospitalization and outpatient management for pregnant women with pregestational and gestational diabetes or mild hyperglycemia;
- To estimate the social profitability ratio of treating pregestational and gestational diabetes and mild hyperglycemia at CIDP-HC-FMB-Unesp.

## METHODS

This study was approved by the Research Ethics Committee of Botucatu Medical School (Faculdade de Medicina de Botucatu, FMB), Unesp, under file no. 493/2007.

### Study design and setting

HC-FMB-Unesp is a general, public, university hospital with 415 beds. It provides secondary and tertiary care, and its obstetrics outpatient unit and maternity unit assist high-risk pregnant women.

CIDP-HC-FMB-Unesp adopts the following protocol: pregnant women with pregestational and gestational diabetes or mild hyperglycemia treated with diet therapy are followed in an outpatient setting; women with pregestational and gestational diabetes or mild hyperglycemia treated with diet plus insulin receive in-hospital care. 

Direct, indirect and total costs, along with benefits, were prospectively estimated for all pregnant inpatients and outpatients with pregestational and gestational diabetes or mild hyperglycemia, and for their babies. These women started prenatal care in 2007 at CIDP-HC-FMB-Unesp.

The subjects were allocated to one of two groups: inpatients - women treated with diet plus insulin managed through hospitalization (n = 20); and outpatients - women treated with diet alone on an outpatient basis (n = 30).

### Treatment

Pregnant women with pregestational and gestational diabe tes or mild hyperglycemia were initially treated with a 2300 calorie diabetic diet individualized to each person’s needs and distributed in seven meals. The women who remained eugly cemic after receiving this type of treatment were followed up on an outpatient basis until delivery, while those showing high glucose levels during the day despite the diet therapy, receivedin-hospital care for one day (24 hours) at fortnightly intervals until 28 weeks of gestation, and at weekly intervals from week 28 until delivery.

### Exclusion criteria

Women with pregestational and gestational diabetes or mild hyper glycemia who developed clinical problems during gestation or did not give birth at our service were excluded from the study.

### Cost calculation

Data were collected longitudinally from each patient’s hospital records, from the time of her first visit or admission until the postpartum examination. These data included information on the clinical and obstetric history, laboratory tests and imaging examinations performed, medication prescribed and supplies used. The data on neonatal care included the following infor mation on the newborn: clinical history, gestational age at birth (weeks), term or preterm, ponderal index, Apgar index, birth weight, birth weight classification according to gestational age (adequate for gestational age = AGA, small for gestational age = SGA, or large for gestational age = LGA), type of care (room ing-in, nursery or neonatal intensive care unit [ICU]) and num ber of days in hospital. The costs extracted from the hospital records, as well as those determined using the full costing method, were separated into direct costs (medication, laboratory tests, imaging examinations, supplies, telephone services and personnel) and indirect costs (water supply, sewage services, power supply, general adminis tration and cleaning services)^21^^,^^22^^,^^23^^,^^24^^,^^25^^,^^26^^,^^27^ (Table 1). All costs were expressed in United States dollar values for the year 2009 (US$ 1.00 = R$ 1.97).

**Table 1.** Mean unit cost and total cost of prenatal care, delivery care, hospitalization for childbirth and postpartum care, neonatal care cost, and total investment cost in United States dollars, for pregnant women with diabetes or mild hyperglycemia subjected to hospitalization or outpatient management at CIDP HC-FMB-Unesp, 2007

Costs	Diabetic inpatients		Diabetic outpatients	
	Cesarean delivery	Vaginal delivery	Cesarean delivery	Vaginal delivery
Prenatal care cost per unit	187.84		53.43	
Prenatal care total cost	2,160.18		341.95	
Cost of delivery	349.10	341.02	349.94	340.52
Unit cost of hospitalization for childbirth and puerperium	189.12	182.26	179.39	173.36
Weighted average cost of hospitalization for childbirth and puerperium	187.06		175.77	
Total cost of hospitalization for childbirth and puerperium	907.80	565.03	789.34	606.76
Total cost of hospitalization (during childbirth and puerperium and for childbirth)	1,256.91	906.05	1,139.28	947.28
Total cost of prenatal care + total cost of hospitalization (during childbirth and puerperium and for childbirth)	3,417.10	3,066.23	1,481.24	1,289.24
Weighted average cost of prenatal care + weighted average cost of hospitalization	3,311.84		1,366.04	
Cost of hospitalization per newborn:				
Rooming-in	6.61		6.61	
Nursery	80.04		80.04	
Neonatal ICU	281.13		281.13	
Total cost of infant hospitalization:				
Rooming-in	26.49		23.63	
Nursery	509.85		493.04	
Neonatal ICU	5,903.77		2,811.31	
Final weighted average cost of neonatal care	513.88		210.57	
Final weighted average cost of maternal and neonatal care	3,825.73		1,576.62	
Total investment cost	76,514.62		47,298.57	

### Pharmacoeconomic assessment of treatment: building decision trees

Treatment success was assessed on the basis of the following maternal and perinatal parameters: mean glycemia ≤ 120 mg/dl, vaginal delivery, gestational age at birth ≥ 37 weeks, weight adequate for gestational age (AGA), ponderal index < 2.5,^28^ infant rooming-in, no maternal mortality and no perinatal mortality.

After the maternal and perinatal criteria for treatment success had been defined, each pregnant woman was classified in accordance with an eight-point scoring system (≥ 6 [equivalent to 75%] = successfully treated; and < 6 = unsuccessfully treated).

For both management methods (outpatient and inpatient), the following were identified: 1) total costs of maternal care (prenatal, childbirth and puerperal) and neonatal care for pregnant women who were successfully or unsuccessfully treated; 2) difference in costs between the treatments. These estimates were used to build a decision tree on the alternatives for managing diabetic pregnant women and their infants.

### Benefit calculation

Benefits in monetary units were divided into two subsets: direct benefits and indirect benefits.^11^ The direct benefits included the expenditures avoided, according to the maternal and perinatal cri teria for successful treatment of diabetes and mild hyperglycemia. The direct benefits in each group were calculated based on the fol lowing parameters: a) maternal - cost of disease treatment, differ ence between successful and unsuccessful treatments, number of hospital days and/or visits during pregnancy or childbirth and dif ference between Cesarean delivery and vaginal delivery costs; and b) neonatal - number of hospital days for the infant and type of care provided (rooming-in, nursery or neonatal ICU). The mone tary values relating to ICU use were calculated based on the mean rates of infants admitted to the neonatal ICU: 16.0% (15.0 and 17.0%) for infants born to diabetic inpatients; and 3.95% (2.9 and 5.0%) for infants born to diabetic outpatients.^8^^,^^21^

The indirect benefits were estimated by considering the increase in social productivity that results from low maternal and perinatal mortality. To calculate the indirect benefits, the maternal and perinatal mortality rates were considered to be 2%^21^ and 4.5% (2.8-6.2%),^22^^,^^23^^,^^24^^,^^25^^,^^26^^,^^27^ respectively. In addition, data from the latest Brazilian census^29^ were used to determine the per capita income in the area of Botucatu, state of São Paulo, which was found to be US$ 216.34/month. The average num bers of years of productive life were 60 years for women and 65 years for men. Thus, an average value of 62.5 years was used to determine the number of labor years earned per newborn. The mean age was 31 years among diabetic inpatients, and 32 years among diabetic outpatients.

To calculate the maternal and perinatal direct benefits (DB) and indirect benefits (IB), the following formulae were constructed:


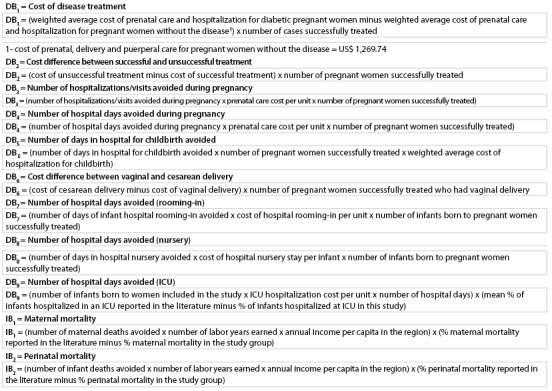


The total cost of the benefits was estimated as the sum of all the direct and indirect benefits.


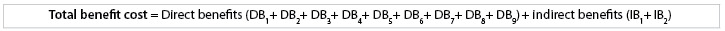


The investment cost was calculated as the sum of the final average costs of maternal and neonatal care.


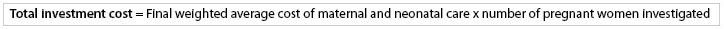


The cost-benefit relationship was determined as the dif ference between the total benefit cost and the total investment cost.^9^


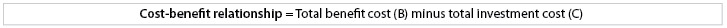


The social profitability ratio was estimated based on the relationship of the benefit/cost difference divided by cost.^11^ A value greater than one indicated that the social value of the benefits exceeded the social value of the costs and that the project/program showed a positive benefit.^13^


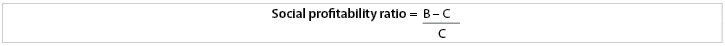


## RESULTS

### Treatment benefits

The maternal and perinatal outcomes are shown in Table 2. The mean maternal glycemia, cesarean delivery rate and ponderal index in infants born to mothers with diabetes or hyperglycemia were higher among inpatients than among outpatients.

The maternal age, gestational age, weight, Apgar index, length, gestational age at birth, length of newborn, hospitalization, type of assistance, newborn classification according to gestational weight/age, maternal mortality and perinatal mortality did not differ significantly between inpatients and outpatients.

**Table 2.** Perinatal and maternal incomes (median/quartile and percentage) observed among pregnant inpatients and outpatients with diabetes or mild hyperglycemia at the Center for Investigation of Perinatal Diabetes, HC-FMB-Unesp, 2007

Variables	Diabetic inpatients (n = 20)	Diabetic outpatients (n = 30)	P
Maternal age (years)	31.0 (26.0;35.0)	32.0 (25.0;37.0)	0.613^*^
Mean glycemia (maternal)	157.0 (121.1;197.9)	97.6 (92.2;108.3)	< 0.001^*^
Maternal mortality	0	0	1.0^†^
Perinatal mortality	0	0	1.0^†^
Gestational age (weeks)	37.5 (36.0;38.0)	38.0 (37.0;40.0)	0.078^*^
Length (cm)	48.3 (45.3;51.0)	50.0 (48.0;51.0)	0.094^*^
Weight (g)	3,385.0 (3062.5;3816.3)	3,470.0 (3055.0;3901.3)	0.797^*^
Ponderal index	3.1 (2.8;3.5)	2.7 (2.5;2.9)	0.010^*^
Apgar at 5 minutes	9.0 (9.0;10.0)	9.0 (9.0;10.0)	0.258^*^
Days of infant hospitalization	4.0 (3.5;8.0)	4.0 (3.0;6.0)	0.322^*^
%			
Cesarean delivery	70.0	40.0	0.015^‡^
Classification according to weight:			
AGA	65.0	63.3	1.0^†^
SGA	0.0	3.3	
LGA	35.0	33.3	
Preterm infant	30.0	10.0	0.130^†^
Term infant	70.0	90.0	
Type of hospital care:			
Rooming-in	55.0	76.7	0.283^†^
Nursery	40.0	20.0	
ICU	5.0	3.3	

^*^Mann-Whitney test; ^†^Fisher’s exact test; ^‡^Chi-square test

AGA = adequate for gestational age; SGA = small for gestational age; LGA = large for gestational age; ICU = intensive care unit

### Decision tree and treatment/social profitability cost-benefit assessment

**Figure 1.**
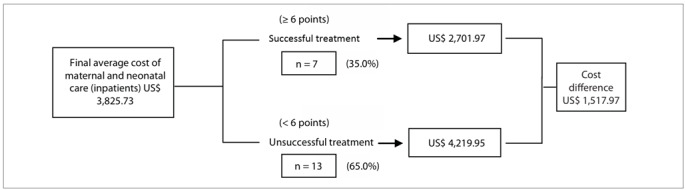
Decision tree for the type of treatment offered to pregnant inpatients with diabetes or mild hyperglycemia.

**Figure 2.**
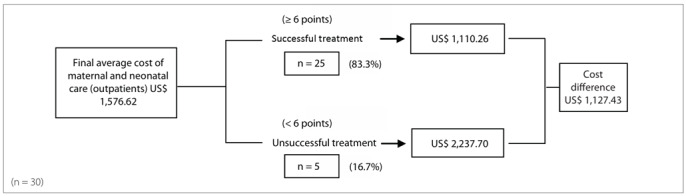
Decision tree for the type of treatment offered to pregnant outpatients with diabetes or mild hyperglycemia.

Decision trees (Figures 1 and 2) showed that for 35.0% (7/20) of the inpatients with diabetes or mild hyperglycemia, the treatment was considered successful. The final weighted average cost of maternal and neonatal hospitalization was US$ 2,701.97, which was a saving of US$ 1,517.97 in comparison with unsuccessful treatment. Among the outpatients with diabetes or mild hyperglycemia, treatment was successful in 83.3% (25/30) of the cases, with a final weighted average cost for maternal and neonatal care of US$ 1,110.26, which was a saving of US$ 1,127.43 in comparison with unsuccessful treatment. 

Table 3 shows that in the group of inpatients successfully treated, compared with those unsuccessfully treated, the following reductions were observed: 2.09 in the number of hospitalizations during pregnancy; 0.48 days in the number of days in hospital during pregnancy; 1.86 days in the number of hospitalizations for childbirth; 3.1 days in infant rooming-in stays; and 2.71 days in infant nursery stay. In addition, 71.4% (5/7) of the women had a vaginal delivery, 85.7% (6/7) of the infants received rooming-in care, 14.3% (1/7) stayed in the hospital nursery and none required neonatal ICU care. 

In the group of successfully treated outpatients, compared with those unsuccessfully treated, the number of visits during gestation had a reduction of 0.2, the number of days of hospitalization for childbirth had a decrease of 1.56 days and infant rooming-in stays were 0.22 days shorter, although the number of days that infants spent in the hospital nursery increased by 2.33 days. In addition, 72.0% (18/25) of the women had a vaginal delivery and 88.0% (22/25) of the infants received rooming-in care, while 12.0% (3/25) were kept in the hospital nursery (Table 3).

The total benefits (maternal and neonatal) in the inpatient group and outpatient group were US$ 220,233.78 and US$ 300,565.80, respectively, while the direct benefits were US$ 44,085.57 and US$ 37,901.12, and the indirect benefits were US$ 176,148.24 and US$ 262,664.68, respectively (Table 4).

The cost-benefit relationships for care were US$ 143,719.16 for diabetic inpatients and US$ 253,267.22 for outpatients, thus resulting in social profitability ratios of 1.87 and 5.35, respectively (Table 5). 

**Table 3.** Distribution of hospitalizations/visits by pregnant inpatients and outpatients with diabetes or mild hyperglycemia and their infants, according to successful and unsuccessful treatment at the Center for Investigation of Perinatal Diabetes, HC-FMB-Unesp, 2007

	Diabetic inpatients			Diabetic outpatients		
	Treatment successful n = 7	Treatment unsuccessful n = 13	Unsuccessful minus successful	Treatment successful n = 25	Treatment unsuccessful n = 5	Unsuccessful minus successful
Average number of hospitalizations/visits (days) during pregnancy	10.14	12.23	2.09	6.4	6.6	0.2
Number of hospital days during pregnancy	1.63	2.11	0.48	-	-	-
Number of hospital days for childbirth	3.14	5.00	1.86	3.64	5.2	1.56
Number of infant hospital days:						
Rooming-in	3.33	6.4	3.1	4.22	4.0	0.22
Nursery	4.0	6.71	2.71	7.33	5.0	- 2.33
ICU	0	21		0	10	
Number of vaginal deliveries	5	1	-	18	0	-
Number of cesarean deliveries	2	12	-	7	5	-
Number of infants:						
Rooming-in	6	5	-	22	1	-
Nursery	1	7	-	3	3	-
ICU	0	1	-	0	1	-
Total	7	13	-	25	5	-

ICU = intensive care unit.

**Table 4.** Benefits resulting from treatment for diabetic inpatients and outpatients at the Center for Investigation of Perinatal Diabetes, HC-FMB-Unesp, 2007

Benefits	Diabetic inpatients (n = 7)	Diabetic outpatients (n = 25)
Direct benefits^*^		
*Maternal:*		
DB1 - disease treatment	14,294.74	2,407.61
DB2 - difference in cost between successful and unsuccessful treatment	10,625.85	28,185.78
DB3 - number of hospitalizations/visits avoided during pregnancy	2,748.13	267.15
DB4 - number of hospital days avoided during gestation	631.14	-
DB5 - number of hospital days avoided during childbirth	2,435.59	6,855.09
DB6 - difference in cost between cesarean and vaginal delivery	40.43	169.58
*Infant:*		
DB7 - number of infant hospital days avoided (rooming-in)	104.42	27.17
DB8 - number of infant hospital days avoided (nursery)	216.90	559.48
DB9 - ICU stay^†^	12,988.29	548.20
Total direct benefits	44,085.57	37,901.12
Indirect benefits^*^		
*Maternal:*		
IB1 - Maternal mortality^‡^	30,115.24	43,615.18
Infant:		
IB2 - Perinatal mortality^§^	146,032.99	219,049.49
Total indirect benefits	176,148.24	262,664.68
Total benefits (US$)^*^	220,233.78	300,565.80

^*^Cost in United States dollars;^†^Considering infant ICU hospitalization rate to be 11.0% (16.0% - 5.0%) for the inpatient group and 0.65% (3.95% - 3.3%) for the outpatient group; ^‡^Considering maternal mortality rate to be 2%^21^; ^§^Considering average perinatal mortality rate to be 4.5% (2.8 - 6.2%)^23^^,^^24^^,^^25^^,^^26^^,^^27^; ICU = intensive care unit.

**Table 5.** Cost-benefit relationship of treatment for diabetic inpatients and outpatients at the Center for Investigation of Perinatal Diabetes, HC-FMB-Unesp, 2007

	Diabetic inpatients	Diabetic outpatients
Investment cost (C)	76,514.61	47,298.57
Benefit (B)	220,233.78	300,565.80
Cost Benefit (B-C)	143,719.16	253,267.22
Social profitability ratio for investment (B - C)/C	1.87	5.35

*Cost in United States dollars

## DISCUSSION

The analyses on both the inpatient and the outpatient care provided to pregnant women with diabetes or mild hyperglycemia revealed that the benefits in monetary units were greater than the investment costs. The cost-benefit analysis indicated that managing diabetic pregnant women on an outpatient basis was economically more advantageous than hospitalization and should be encouraged.

In cost-benefit analysis, all the outcomes are measured in monetary units, so that any health benefits are translated into economic terms. This method is popular and preferred in other economic sectors because it clearly determines whether a new type of technology saves money and simplifies policy decision-making. However, because this type of analysis requires placing a monetary value on a human life, cost-benefit analyses have not been performed very often for healthcare.^14^

To determine whether treating pregnant women with diabetes or mild hyperglycemia provided sufficient benefits to justify its cost, certain steps and criteria were followed. The first step was to define treatment success. Thus, successful treatment was determined based on the recommendations of the St. Vincent Declaration^30^ and the criteria of the American Diabetes Association (ADA).^31^ The maternal outcomes obtained in this study confirmed that glucose control was more difficult to achieve in diabetic women treated with diet and insulin therapy, who still showed higher glycemic levels despite strict control and treatment. However, the only unfavorable perinatal outcome that correlated with a slightly higher maternal glycemic mean was higher ponderal index. No significant difference was observed among any of the other perinatal parameters. Treatment success is the main goal of prenatal care for diabetic pregnant women. However, besides the treatment itself, successis also related to patient compliance^32^ and the absence of repercussions from hyperglycemia on the placenta.^33^ The rate of treatment success involving maternal and perinatal outcomes was much higher when diet therapy alone was used. The final average cost of maternal and neonatal care was higher for diabetic inpatients, but treatment was successful at a lower cost in both the hospitalization and the outpatient care groups. The analysis on the decision tree confirmed that treatment success among pregnant women with diabetes and mild hyperglycemia was associated with cost reduction, regardless of management method (inpatient or outpatient) and treatment type (diet or diet + insulin).

Successful in-hospital treatment saved US$ 1,517.97 in comparison with unsuccessful treatment, accounting for a 35.97% reduction in total care expenditure. In the group of pregnant women managed in an outpatient setting, successful treatment avoided US$ 1,127.43 in costs, in comparison with unsuccessful treatment. This represented a 50.38% reduction in total care cost.

In both the inpatient and the outpatient groups that were successfully treated, the number of hospitalizations/visits during pregnancy was reduced; and the length of hospitalization (in days) during pregnancy and childbirth and the infant stay in the neonatal ICU were shorter. However, shorter stays of infants rooming in or receiving nursery care were only observed in the group of diabetic inpatients.

According to Nachum et al.,^8^ the advantages of outpatient management over hospitalization for pregnant women with diabetes are that it avoids the psychological, mental and social trauma that may result from in-hospital care, prevents disruption of the family unit and enables the patient to continue working. Home glucose monitoring reflects true ambient glycemic control, and the patient plays an active role in caring for herself. Moreover, these authors reported that all these advantages are associated with a reduction in the costs of diabetes in pregnancy, and concluded that although these results should be interpreted with caution, outpatient care is as effective as hospitalization for pregnant diabetic women, who benefit from convenience and lower hospital costs.

The analysis on the investment made by the CIDP of HC-FMB-Unesp, regarding different types of care, showed that among pregnant women with diabetes or mild hyperglycemia managed on an outpatient basis, treatment success rates were higher than among those managed by hospitalization. Both the direct and the indirect maternal and neonatal benefits demonstrated that pregnant women with diabetes or mild hyperglycemia treated with diet and insulin therapy were less likely to achieve glycemic control, and therefore benefited less than those managed in an outpatient setting. In consequence, the cost-benefit ratio was lower for diabetic inpatients treated with diet plus insulin. The same occurred regarding the social profitability ratio for the investment.

The costs relating to pregnant inpatients with diabetes or mild hyperglycemia were higher than the costs for such patients treated as outpatients. However, the neonatal outcomes were similar to those observed in the outpatient group, except for the glycemic mean, ponderal index and cesarean delivery rate. These are important findings. Among pregnant women managed through hospitalization, the form of the disease is usually more complex clinically, adequate glycemic control is less likely to be achieved during pregnancy and insulin is required to control maternal glycemia. The decision tree confirmed that treatment success avoided additional costs. The benefits in monetary units were greater than the investment made by the CIDP of HC-FMB-Unesp in different types of care for pregnant women. Outpatient management was economically more advantageous. In addition, it also offered other potential benefits such as preservation of the family unit, avoidance of psychological, mental and social trauma resulting from hospitalization, ability to continue with daily activities and active participation in treatment. Physicians not only need to understand the risks, benefits and economics of treatment, but also must carefully select who to treat, when to treat and for how long to do so,^14^ given that as well as being patient advocates, they are also arbiters of societal resources. The results from this study show that the treatment for diabetic pregnant women should be randomized in order to avoid the bias of previously classifying maternal risk and, as a result, to enable management of the most severe cases through frequent short hospitalizations. Using randomization to determine management method so that cost-effectiveness can also be estimated is the next follow-up step to the present study.

## CONCLUSIONS

This study made it possible to identify the direct, indirect and total costs and the benefits of prenatal care, delivery care and neonatal care provided by the CIDP of HC-FMB-Unesp.

The maternal and perinatal criteria for treatment success were mean glycemia ≤ 120 mg/dl, vaginal delivery, gestational age at birth ≥ 37 weeks, weight adequate for gestational age (AGA), ponderal index < 2.5, infant rooming-in, no maternal mortality and no perinatal mortality. The decision tree confirmed that treatment success avoided additional costs.

Cost-benefit analysis showed that outpatient management was economically more advantageous than hospitalization. The social profitability of both treatments was greater than one, thus demonstrating that both types of care for diabetic pregnant women had positive benefits.
